# Influence of Preloading on Damage in CFRP Composite Material Subjected to Low-Energy Impact Loads

**DOI:** 10.3390/ma18174016

**Published:** 2025-08-27

**Authors:** Piotr Arkuszyński, Marek Rośkowicz

**Affiliations:** Faculty of Mechatronics, Armament and Aerospace, Military University of Technology, 00-908 Warszawa, Poland; marek.roskowicz@wat.edu.pl

**Keywords:** composite material, CFRP, material pre-stress, impact loads, impact

## Abstract

A major challenge in the operation of aerostructures made of carbon fiber-reinforced polymer (CFRP) composites is their vulnerability to impact-induced damage, particularly when access is limited to only one side of the affected structure. This issue becomes even more complex considering that damage typically occurs in components subjected to initial static preloading. The objective of this study was to investigate the influence of static preload on the extent of damage in CFRP composites subjected to impact energy and to evaluate the effectiveness of selected non-destructive testing (NDT) methods in damage detection. Experimental investigations were conducted on a series of CFRP specimens impacted using a drop-weight tower. Each impact event was recorded with a high-speed camera operating at ultra-high frame rates. It was demonstrated that both the contact time between the impactor and the specimen, as well as the extent of internal damage assessed using ultrasonic testing (UT) and computed tomography (CT), were significantly affected by the level of applied static preload.

## 1. Introduction

In modern aircraft construction, conventional materials—primarily aluminum alloys of the 2000 series—are increasingly being replaced by materials offering superior mechanical and functional properties [1,2,3]. Among the most technologically advanced representatives of this group are carbon fiber-reinforced polymer (CFRP) composites. These materials, in addition to their favorable strength-to-weight and stiffness characteristics, also exhibit high corrosion resistance and excellent fatigue performance [4,5,6].

One significant drawback of CFRP composites, however, is their relatively low tolerance to impact loads compared to metallic materials. This limitation arises mainly from the anisotropic and layered nature of the composite, which lacks fiber reinforcement in the through-thickness (stacking) direction. As a result, CFRP structures are particularly susceptible to impact-induced damage from both high- and low-velocity impacts [7,8].

The issue of impact loading of composite material was presented by Abrate [9], using ultrasonic testing, evaluating the empirical relationship between the initial kinetic energy of the impactor and the area of damaged material. In his research, he showed that the extent of damage increases with impact energy but there is no clear threshold of impact energy for damage initiation. Reid, Zhou, and Güneş [10,11] studied the effect of impact loading of a composite material on its degradation and defined the resulting damage by the laws of fracture mechanics. They found that the energy required for the initiation of interlaminar damage was lower compared to the potential energy required for fracture of the material along the fibers. In addition, using non-destructive testing, they demonstrated a relationship between the type of composite material and surface finish and its behavior during impact loading.

In aerospace operations, low-velocity impact (LVI) represents a more challenging form of damage. Unlike high-velocity impacts, which typically generate visible and easily detectable surface damage during standard inspections, LVI events—caused by objects traveling at velocities below approximately 10 m/s—often result in internal degradation that is not apparent from the impacted side. This type of damage is commonly referred to as Barely Visible Impact Damage (BVID) [12,13,14].

BVID typically occurs in the form of matrix cracking and localized interlaminar delamination, which significantly degrade both the stiffness and strength of the composite structure [15]. As a consequence, the load-bearing capacity of the affected component may be substantially reduced, potentially leading to premature buckling under compression or failure under service loads lower than the design threshold. Additionally, such internal flaws can act as initiation sites for secondary damage, further reducing the fatigue life of the component under cyclic loading conditions [16,17,18,19].

Damage assessment of dynamically loaded composite materials was also performed under static pre-stressing conditions. It is important to note that airframe structures are continuously subjected to static preloading arising from factors such as fuel weight and aerodynamic forces. Experimental studies on pre-stressed composites subjected to subsequent low-velocity impact loading have been conducted by Sun et al. [20], Malekzadeh [21], Khalili [22], and Choi [23], among others. These researchers performed comparative analyses of composite specimens tested with and without an initial tensile preload.

The results indicate that an increase in initial tensile stress leads to a higher recorded contact force during impact; however, it simultaneously causes a decrease in both the contact time between the impactor and the specimen and the specimen’s maximum deflection. This reduced contact duration shortens the period of stress transmission within the material, thereby diminishing the extent of damage generated in the composite.

Furthermore, it has been observed that a preload applied transversely to the fiber orientation has a more pronounced effect on the level of induced damage than a preload applied parallel to the fiber direction. Abrate [24] also investigated preloaded composite specimens and studied the progression of delamination using ultrasonic C-scan inspection techniques [25]. He demonstrated that a specific threshold preload exists, beyond which delamination is initiated during low-velocity impacts. Knowledge of this critical load value enables designers to assess structural vulnerability and predict the behavior of impacted composite materials under operational conditions.

Low-velocity impact tests have also been performed on composite specimens subjected to biaxial preloading. The results reported by Gracia-Castillo et al. [26] indicated that the influence of biaxial preload on damage development is comparable to that of uniaxial preload. However, the orientation of the applied preload with respect to the fiber direction plays a crucial role in damage formation; whether the load is aligned parallel or perpendicular to the fiber architecture directly affects the extent of the resulting damage. Similar conclusions were drawn by Mitrevski [27], who investigated the response of thin glass fiber-reinforced polymer (GFRP) composites under biaxial preloading, additionally found that increasing the preload reduced both the contact duration between the impactor and the specimen and the overall deflection, thereby reducing the extent of impact-induced damage.

Low-velocity impact tests on composite structures subjected to different types of preloading—tensile, compressive, and shear—have also been presented in the works of Robb [28], Chiu [29], Zhang [30] and McCallum [31]. Their findings revealed that compressive preloading resulted in larger damage areas compared to tensile or shear loading. Whittingham [32], based on impact experiments with preloaded specimens, concluded that for low-energy impacts (6 J), the depth of matrix cracking and the absorbed energy were largely independent of the type and magnitude of preload. However, at higher energy levels (10 J), the preload significantly influenced the extent of damage. Comparative tests were performed for composites under no preload and under various configurations of uniaxial (tensile/compressive) and biaxial (tension/tension, compression/compression, tension/compression) preloading.

Dynamic tests on preloaded composite materials have also been extended to high-velocity impact scenarios. Herszberg and Lan [33,34,35] conducted experimental studies on CFRP composites impacted at velocities up to 70 m/s under tensile and compressive preloading. One of the key observations was that under compressive preload, the velocity threshold required to initiate damage was considerably lower.

The issue of combined static and impact loading of composite materials is highly complex and remains relatively underexplored, despite the growing number of publications on composite failure mechanisms. An important reason to include this factor in strength assessments is the fact that composite components in aircraft structures are routinely subjected to static loads (e.g., due to mass or aerodynamic effects) while also experiencing simultaneous impact loads during service.

In the present study, the influence of the type and magnitude of pre-stress on the extent of damage in a CFRP composite subjected to impact loading was investigated. The experimental methodology involved pre-stressing the specimen through bending, resulting in the simultaneous presence of tensile and compressive zones. Impact loading was applied using a drop-weight impactor, and the extent of damage was evaluated using ultrasonic testing (UT) and computed tomography (CT).

Recent advances in real-time damage tracking of CFRP composites have largely relied on digital image correlation (DIC) [36,37]. The present work extends this research direction by demonstrating the efficacy of high-speed imaging as an alternative experimental approach, enabling the simultaneous characterization of transient impact phenomena and the corresponding force in time responses measured by the instrumented impactor. A further novelty of the proposed approach lies in its experimental simplicity: unlike biaxial loading configurations, which require complex and specialized test setups, four-point bending provides a straightforward and repeatable method for generating a controlled stress state.

## 2. Materials and Methods

Experimental investigations were conducted to assess the damage resulting from impact loading, using computed tomography (CT) and ultrasonic testing (UT) techniques. The study focused on damage occurring in carbon fiber-reinforced polymer (CFRP) materials as a result of low-energy impacts.

The tested specimens were prepared using carbon fiber prepreg GG 204T g/m^2^ IMP 503 ZHT. Each specimen consisted of 25 unidirectional layers, arranged according to the stacking sequence [0°]_25_. The composite laminate was fabricated using an autoclave process at the Silesian Science and Technology Centre of Aviation Industry (Czechowice–Dziedzice, Poland), following the manufacturer’s specifications. The curing process was carried out at a pressure of 400 kPa and a temperature of 120 °C.

In accordance with PN-EN ISO 14125 [38], rectangular specimens with nominal dimensions of 60 mm × 250 mm and a thickness of 7.3 mm were extracted from the fabricated laminate plate using abrasive water-jet (WaterJet, Monza, Italy) cutting technology. The dimensional accuracy achieved through the cutting process was consistently high, allowing the influence of dimensional variability on the test results to be considered negligible.

Impact tests were performed using an Instron Ceast 9440 (CEAST, Pianezza, Italy) drop-weight impact tester, equipped with a force sensor for recording the contact force during impact. Based on preliminary investigations, it was determined that impact tests would be conducted for energy values in the range of 10 J to 40 J, corresponding to the desire to induce Barely Visible Impact Damage (BVID) in the specimens. Higher energy levels were excluded due to complete specimen failure, which occurred regardless of the applied static preload. The selected energy range is consistent with data provided by Boeing for impacts resulting in Barely Visible Impact Damage (BVID) in CFRP structures [39].

**Figure 1:**
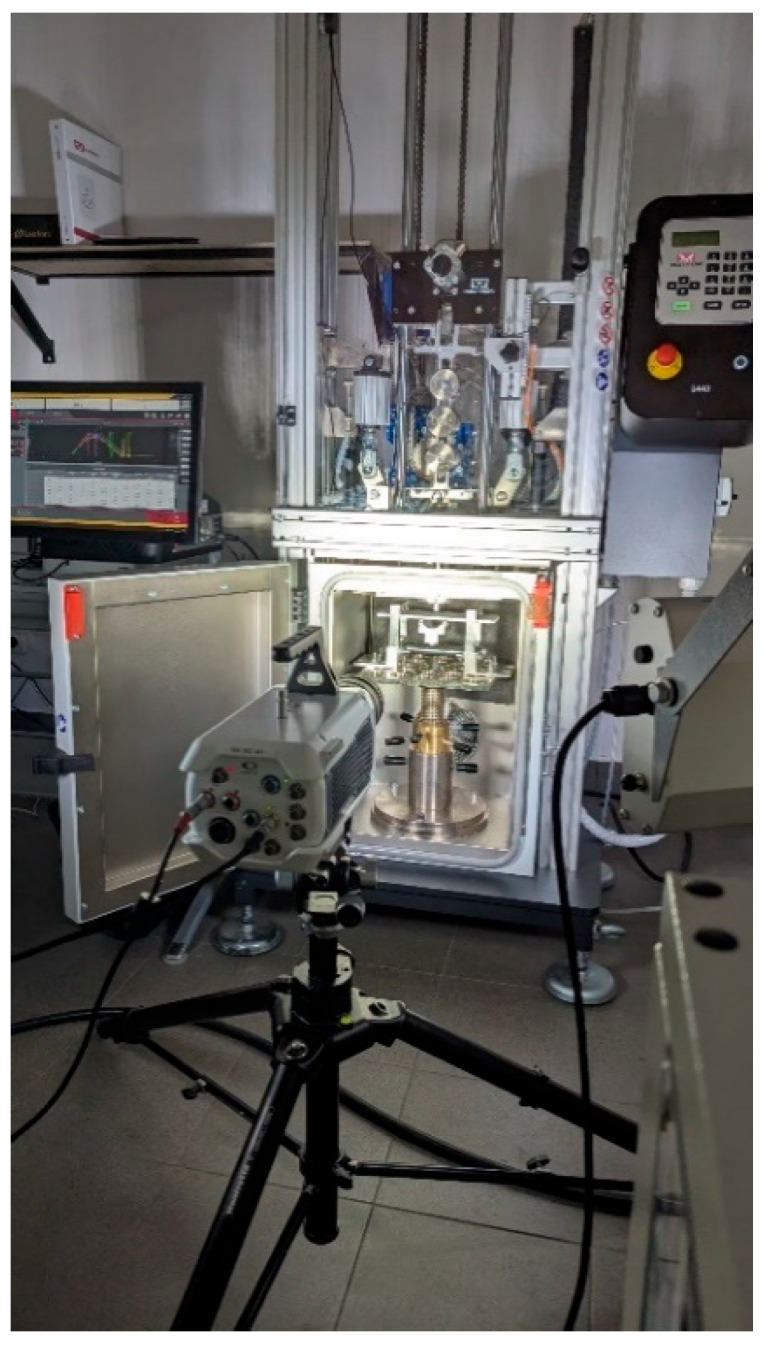
Impact test bench (including high-speed camera).

All impact events were recorded using a Chronos 1.4 high-speed camera operating at 40,413 fps, allowing for detailed observation of the impactor–specimen interaction with high temporal resolution. Figure 1 presents the experimental test setup.

A GE Phoenix v/tome/x m 300 computed tomography (CT) scanner (ITA, Poznań, Poland), with a maximum X-ray source power of 300 kV/500 W, was used to evaluate the post-impact condition of the composite specimens. The CT scans were performed under the following parameters: X-ray tube voltage of 120 kV, current intensity of 120 μA, number of projections ranging from 360 to 1440, and filtration using a combination of 0.5 mm Cu + 0.5 mm filters. The detector panel resolution was 2048 × 2048 pixels.

Ultrasonic examinations were conducted using the Dolphicam 2 system (Dolphitech AS, Gjøvik, Norway) equipped with a 128 × 128 multi-transducer probe head, where each individual transducer measured 0.25 mm. The testing protocol employed 32 active transducers with signal interpolation over 4 transducers. The speed of sound used to calibrate the time base in the composite material was 3070 m/s. All measurements were performed with a 5 MHz probe, referenced to a fixed calibration point.

### 2.1. Four-Point Bending Test and Evaluation of Stiffness Variation in Impacted Specimens Without Preload

In the initial phase of testing, the prepared specimens were subjected to impact loading without pre-stressing the composite material. Impact tests were conducted at energy levels of 10 J, 15 J, 20 J, 30 J, and 40 J. For each impact energy, three specimens were tested.

**Figure 2:**
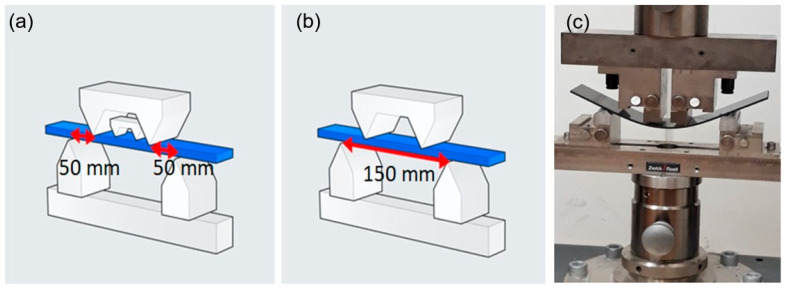
(**a**,**b**) Diagrams of support assembly during four-point bending test of composite material, and (**c**) view of specimen mounted in bending instrument.

To evaluate the effect of impact loading on the mechanical properties of the material, comparative stiffness measurements were performed via four-point bending tests using a Zwick Roell Z100 (Zwick Roell Group, Ulm, Norway) testing machine. Bending tests were conducted both prior to impact loading and after impact testing. The tests followed the setup illustrated in Figure 2, maintaining a support span of 150 mm between the lower supports and 50 mm between the upper loading points (Figure 2a,b). The support configuration was designed in accordance with the requirements specified in the composite bending test standard PN-EN ISO 14125.

The loading applied during the bending tests did not exceed 50% of the failure load. The failure load of the material under bending was determined using a static four-point bending test performed on three randomly selected specimens.

### 2.2. Dynamic Testing of Preloaded Specimens

**Figure 3:**
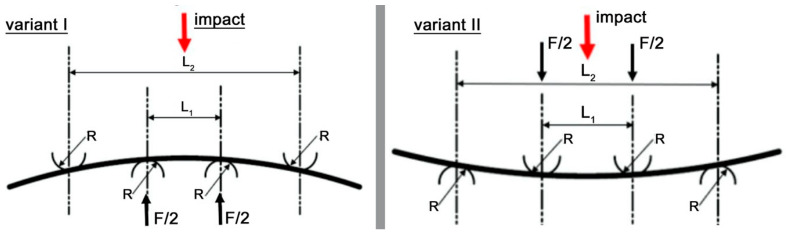
Schematic of preload variants for the composite material.

Pre-stressing of the composite material was performed using a four-point bending setup. Two variants of pre-stressing were considered: in variant I, the impact was applied to the specimen on the tensile fiber side, whereas in variant II, the impact was applied on the compression fiber side (see Figure 3).

This configuration is also relevant from the perspective of service conditions, since in aircraft fuselage skins, particularly in the vicinity of local stiffeners, complex stress states frequently occur, including combined bending and tensile/compressive loading. Moreover, the adopted arrangement facilitates the placement of pre-stressed specimens in a conventional drop-weight tower, thereby enabling the investigation of impact response under controlled pre-stress conditions.

**Figure 4:**
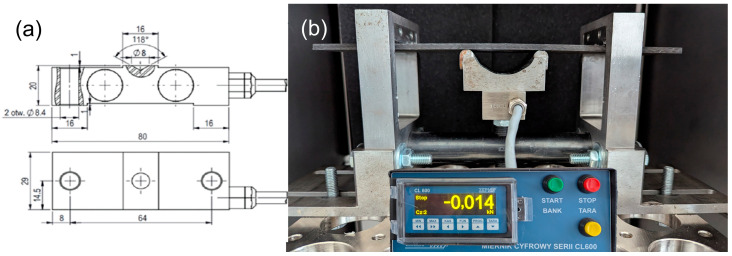
(**a**) Design of the force sensor and (**b**) final assembly of the sensor with integrated force gauge.

To enable the preloading of specimens mounted in these fixtures, a custom force gauge with the geometry depicted in Figure 4 was used, designed and manufactured by ZEPWN J. Czerwiński i Wspólnicy–Spółka Jawna (Marki, Poland).

Figure 4 presents both the design schematic of the sensor and its appearance following manufacture with the installed gauge.

The force gauge, integrated with the measurement system, was employed within the preload fixtures to accurately control and define the applied load. Two separate fixtures were constructed for each preload variant (I and II), differing primarily in the design of the loading element. The preload was applied using a screw mechanism.

**Figure 5:**
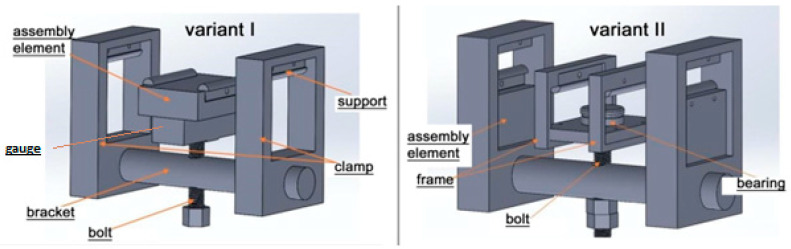
Instrument diagrams for two variants of specimen pre-stressing.

Figure 5 presents views of the fixtures designed for both variants.

**Figure 6:**
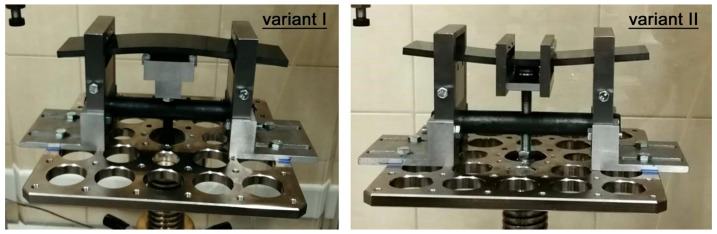
View of specimens pre-stressed (variants I and II) and mounted in the instrument on the drop hammer base.

Pre-stressed specimens mounted in the test fixtures for both variants are presented in Figure 6.

## 3. Results

### 3.1. Four-Point Bending Test and Evaluation of Stiffness Variation in Impacted Specimens Without Preload

**Figure 7:**
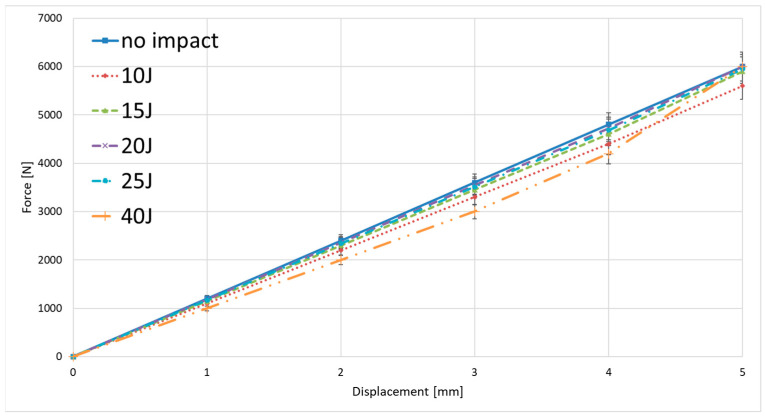
Force–displacement curves of the testing machine crosshead for specimens without dynamic loading and subjected to impact energies ranging from 10 to 40 J.

Examples of static loading curves for the composite material during the bending test—expressed as the variation of force versus crosshead displacement of the testing machine—before and after impact tests are presented in Figure 7. Specimens subjected to impact loading at different impact energies within the range of 10–40 J were used for comparative analysis. As noted previously, the bending load was limited to no more than 50% of the failure load. The failure loads determined from three specimens were 14.144 kN, 14.232 kN, and 14.022 kN, respectively, with a mean value of 14.133 kN. This mean failure load corresponded to a maximum normal (tensile/compressive) stress of approximately 663 MPa in the material.

**Figure 8:**
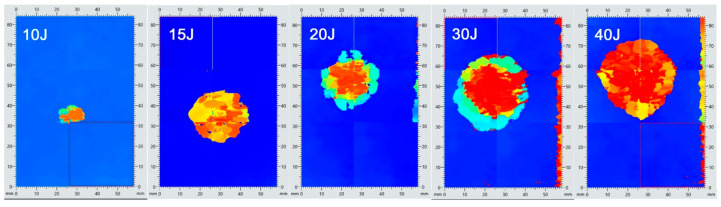
Results of ultrasonic tests of material specimens loaded with energies in the range of 10–40 J.

For impact energies of 10 J to 15 J, changes in stiffness were negligible, indicating an absence of fiber damage, as confirmed by computed tomography (CT) scans. Alterations in the deformation response of the material following impact loading were observed only for specimens subjected to impact energies of 20 J, 30 J, and 40 J. These changes were evidently associated with the geometry of the damage sustained by the material due to impact loading. An example of ultrasonic testing results, with the damage areas highlighted for specimens impacted at different energy levels, is presented in Figure 8.

**Figure 9:**
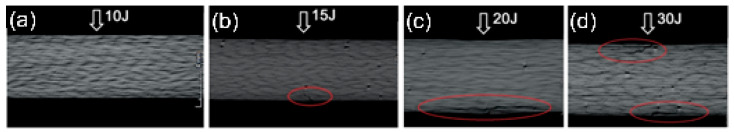
Tomograms of the cross-section of specimens loaded with energy. (**a**) 10 J, (**b**)15 J, (**c**) 20 J, and (**d**) 30 J.

As expected, a distinct trend was observed: with increasing impact energy, the extent of damaged material also increased. In addition, the location of the damage changed—higher energy levels resulted in damage zones occurring deeper beneath the surface of impactor contact. Changes in the cross-sectional structure of the material following impact loading—such as damage depth and damage morphology—were further evaluated using Computed Tomography (CT). Representative results from this part of the experimental study are presented in Figure 9. The damages in specimens are marked with red circles. 

Loading the material with an energy of 10 J did not result in any visible damage on the tomograms. At an energy level of 15 J, damage was detected in the form of cracks in both the matrix and fibers, as well as local delamination. The damage occurred on the non-impacted side, reaching a depth of approximately 15% of the specimen thickness. For an energy of 20 J, damage of a similar nature was also observed on the unstroked side of the specimen. For an impact energy of 30 J, damage was observed on both the impacted and non-impacted sides, with an estimated penetration depth of approximately 25% of the specimen thickness. It should be emphasized that in none of the cases described above was damage observed in the central part of the specimens’ cross-section.

**Figure 10:**
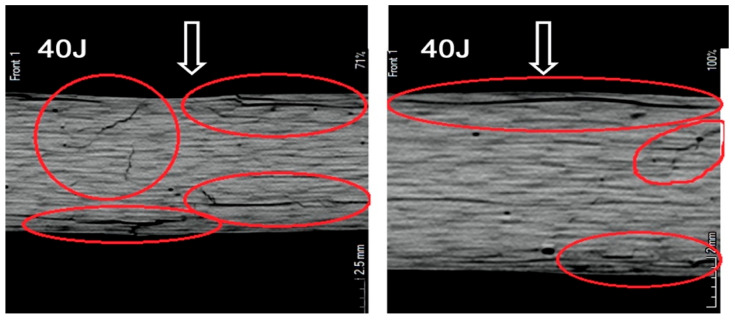
Tomograms of the cross-section of a specimen loaded with an energy of 40 J.

An intensification of the material failure process with increasing impact load was to be expected. The tomograms obtained for an impact energy of 40 J confirmed this assumption. At this energy level, the specimens exhibited through-thickness damage, with numerous cracks and widespread delamination observed throughout the material (Figure 10). The damages in specimens are marked with red circles.

**Figure 11:**
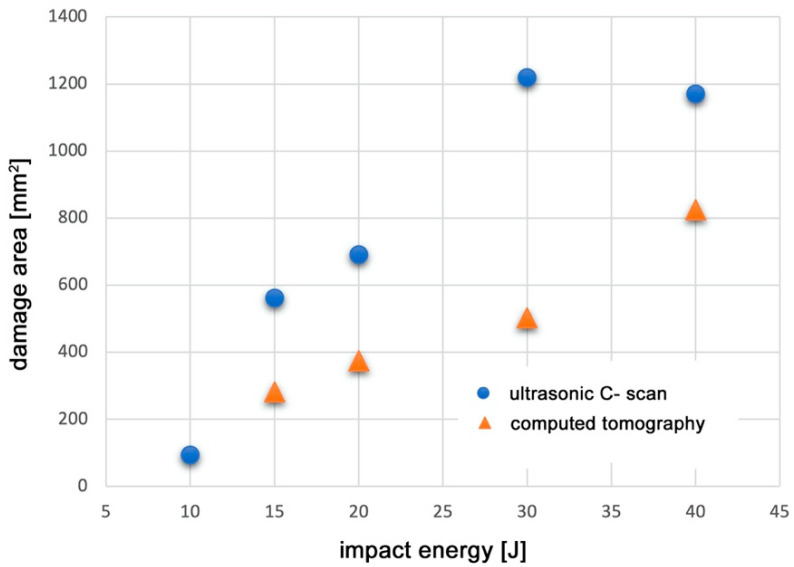
Comparison of damage extent values determined by ultrasonic testing (UT) and computed tomography (CT) scan methods.

Applying the analytical capabilities of the software for processing recorded tomography and ultrasonic images, an additional quantitative analysis was conducted to evaluate the geometry of damage induced in the material as a function of impact energy. The maximum extent of damage within the striker impact zone was assessed using two measurement modalities: ultrasound (UT) and computed tomography (CT). The results of this analysis are presented in Figure 11.

The observed deviation from the expected increasing trend—specifically, the larger defect area measured for the specimen impacted at 30 J compared to 40 J—highlights the lower accuracy of the ultrasonic testing method relative to computed tomography. Considering the higher precision of tomography, a clear correlation was identified: the defect surface area measured by ultrasonic testing tends to overestimate the actual damage size, in some cases by nearly threefold. Nevertheless, due to the intrinsic limitations of the tomography method concerning specimen size, further tests were conducted using ultrasonic testing, which remains the most widely applied technique in the aerospace industry.

Further investigations involving pre-stressed specimens were conducted using an impactor energy of 15 J, as impacts at this energy level resulted in Barely Visible Impact Damage (BVID) for specimens without initial static loading. The impactor energy was selected empirically: lower energy impacts did not induce damage detectable by non-destructive ultrasonic testing, whereas higher energy impacts caused defects visible to the unaided eye. Moreover, at 15 J the damage exhibited the largest projected surface area of BVID in unloaded specimens; for higher energies only, the through-thickness location of damage changed, without a significant increase in the affected surface.

### 3.2. Dynamic Tests on Pre-Loaded Specimens

**Figure 12:**
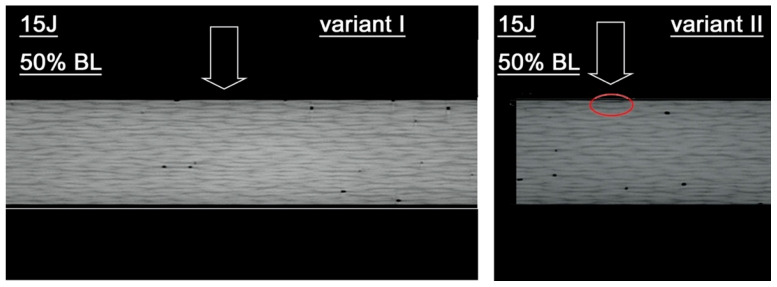
Tomograms of the cross-section of the specimens for two preload variants (variant I, variant II, 50% failure load) subjected to impact loading with an impact energy of 15 J.

Examples of test results for pre-stressed material subjected to 50% of the failure load in variants I and II are presented in Figure 11. In variant I, the impact did not cause any detectable damage to the material. Specimens in variant II displayed limited, localized structural damage. Figure 12 illustrates damage located at a depth of approximately 0.6 mm. The damages in specimens are marked with red circles. The arrow indicates the impact point. 

**Figure 13:**
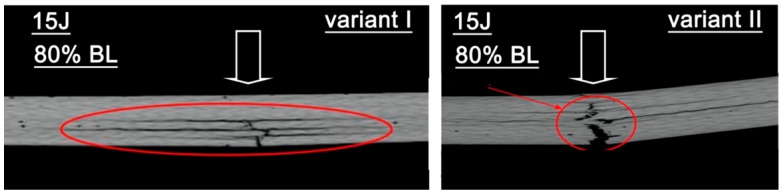
Tomograms of the cross-section of the specimens for two preload variants (variant I, variant II, 80% failure load) subjected to impact loading with an impact energy of 15 J.

Damage caused by the impact on the pre-stressed material subjected to a significantly higher static load (80% of the failure load) is presented in Figure 13. The damages in specimens are marked with red circles. The arrow indicates the impact point.

For pre-stress variant I, tomographic analysis of the material structure after dynamic testing revealed damage extending to approximately half the specimen thickness. The tomograms identified damage in the form of fiber breaks, matrix cracks, and delaminations. Notably, the damage was confined to the side opposite the impactor contact. In variant II, the dynamically loaded specimen exhibited fracture, with delaminations and damage to both fibers and matrix observed throughout the specimen volume.

**Figure 14:**
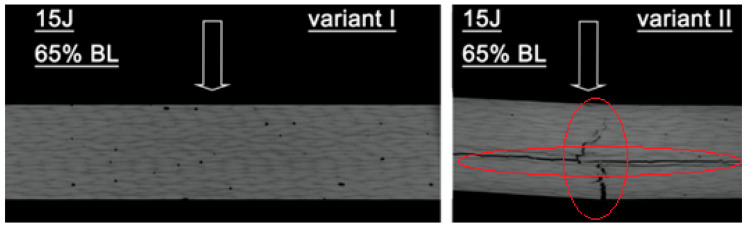
Tomograms of the cross-section of the specimens for two variants of preload (variant I, variant II, 65% failure load) subjected to impact loading with an impact energy of 15 J.

As these observations confirmed a consistent relationship between the magnitude of material damage and the preloading variant, further tests were conducted by adjusting the preload level to 65% of the failure load. An example of the results obtained at this preload level is presented in Figure 14. The damages in specimens are marked with red circles. The arrow indicates the impact point.

For variant I, no damage was detected in the specimen. In contrast, specimens for variant II exhibited significant damage, with defects extending through more than 80% of the material thickness. The observed damage included fiber breakage, delamination, and matrix cracking. The consistency of these results was confirmed through testing on two additional specimens.

**Figure 15:**
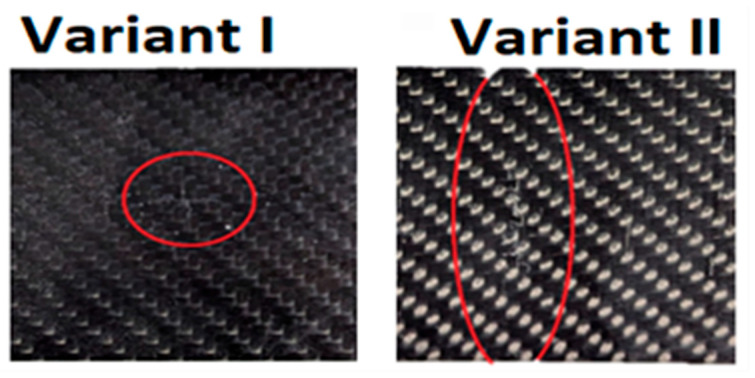
Unaided eye view of damage on the impactor-contacting layer for two preload variant (variant I and variant II at 65% of the failure load) specimens subjected to impact loading with an impact energy of 15 J.

**Figure 16:**
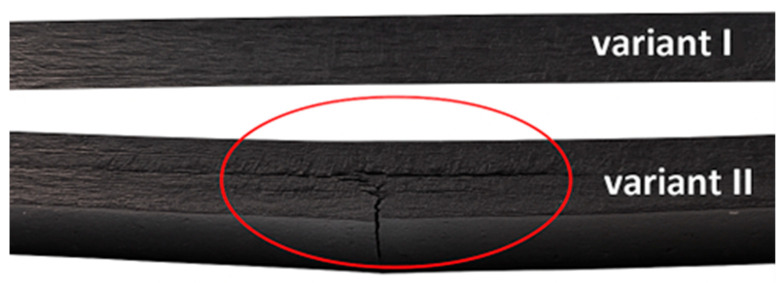
Unaided eye cross-sectional view of the damage in two preload variant (variant I, variant II, 65% failure load) specimens subjected to impact loading with an impact energy of 15 J.

In the case of variant I, a slight surface indentation is visible to the unaided eye. Conversely, in variant II, a deformation indicative of a deep fracture within the material structure can be observed. Figure 15 presents the impact-side views of the specimens from both variants as seen with the unaided eye, while Figure 16 shows cross-sectional views of the same specimens.

**Figure 17:**
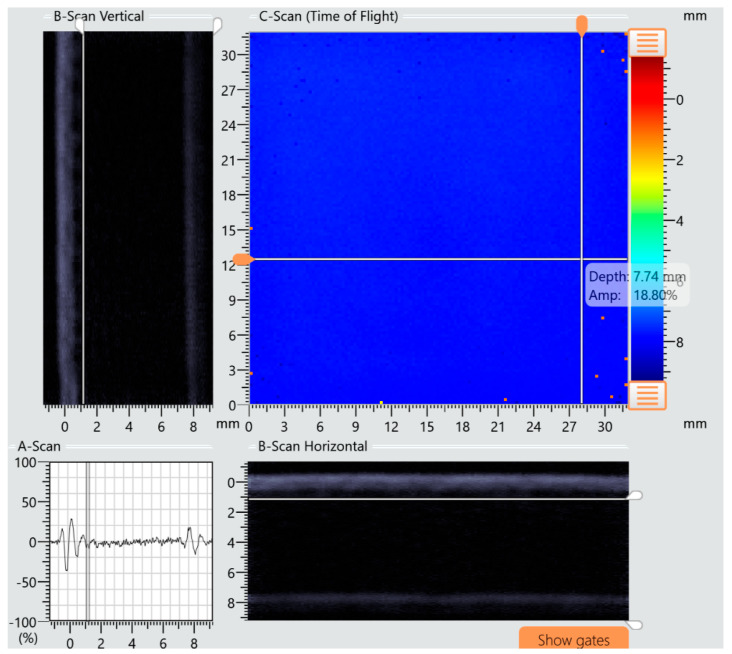
Results of A, B, and C ultrasonic tests of specimens for variant I preload of 65% failure subjected to impact loading with an impact energy of 15 J (no defect).

**Figure 18:**
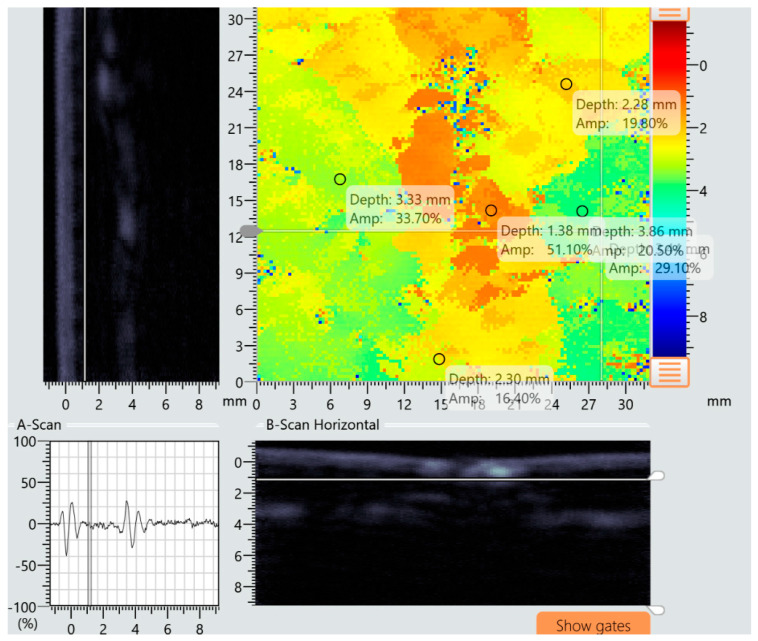
Results of A, B, and C ultrasonic tests of specimens for variant II preload of 65% of the failure load subjected to impact loading with an impact energy of 15 J.

The specimens presented in Figure 15 and Figure 16 were further examined using ultrasonic testing. Analysis of the A-scan, B-scan, and C-scan images confirmed the absence of damage in specimens preloaded according to variant I and the presence of multiple damage zones in specimens subjected to variant II loading. Figure 17 presents a compilation of A-, B-, and C-scan images for the specimen tested under variant I conditions, while Figure 18 shows the corresponding scan results for the specimen tested under variant II conditions. The damages in specimens are marked with red circles. 

**Figure 19:**
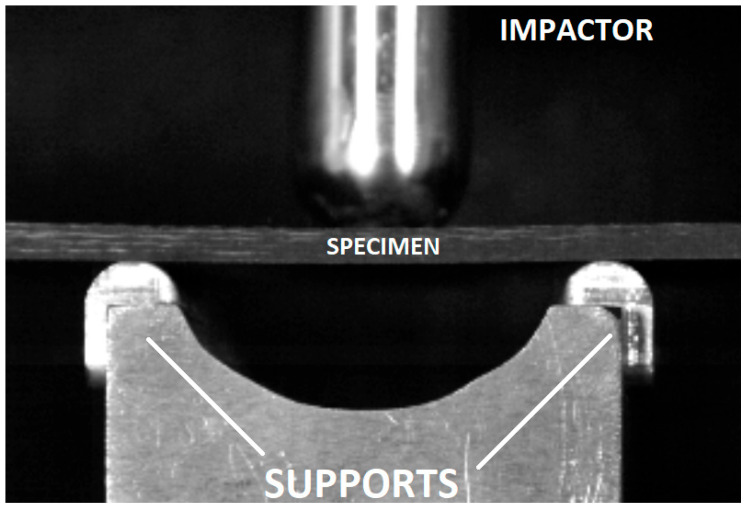
Representative frame captured using the Chronos 1.4 ultra-high-speed camera.

Dynamic impact tests were recorded using a Chronos 1.4 (Kron Technologies Inc, Burnaby, BC, Canada) ultra-high-speed camera. Time-resolved image analysis enabled the estimation of the contact duration between the impactor and the composite specimen. An exemplary frame from the recorded sequence is presented in Figure 19.

Based on the analysis of four specimens exhibiting typical barely visible impact damage (BVID), it was determined that the contact time of the impactor in specimens impacted from the tension side (variant I) was over 30% shorter compared to those impacted from the compression side. The results are summarized in Table 1.

The drop-weight impact tester used in the study was equipped with an instrumented striker. Utilizing the capabilities of the integrated measurement system, the force in time response during impact was recorded and analyzed.

**Figure 20:**
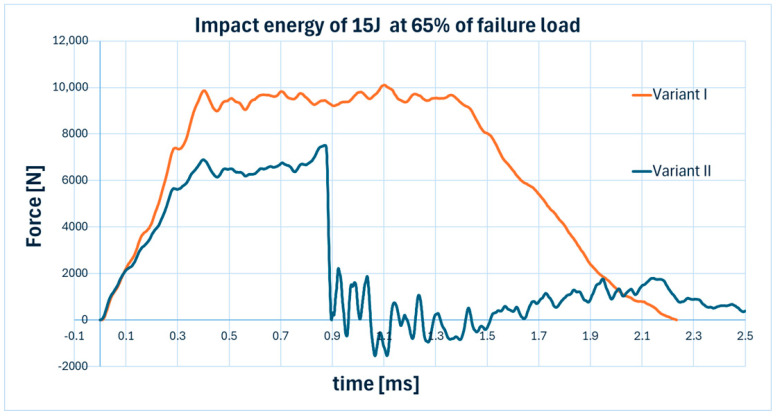
Plot of force in time for an impact energy of 15 J for specimens pre-stressed in two variants at 65% of failure load.

Representative graphs illustrating the variation of impact force over time for an initial material stress equal to 65% of the failure load and an impact energy of 15 J are presented in Figure 20.

Only the variant II plot (blue curve corresponding to the specimen impacted from the compression side) crosses below zero on the vertical axis, indicating a reversal in the direction of the force vector. This behavior confirms that the specimen was fully penetrated.

Analysis of the force–time curves allowed for the identification of damage in the tested specimens. Sudden drops in force typically correspond to material failure (often of the matrix), while a force value falling below zero clearly indicates complete perforation f the specimen [40,41,42].

## 4. Discussion

Experimental investigations demonstrated that, at low impact energies, damage in composite materials can initiate on the side opposite to the striker impact location. This presents a significant challenge, particularly in the structural health monitoring of composite airframes, as the impactor may not leave any visible trace on the surface, making damage detection with the unaided eye problematic.

Changes in the stiffness of the CFRP composite (based on load–displacement curves) were observed only in specimens subjected to impact energies of 20 J, 30 J, and 40 J. For lower energy levels (10 J and 15 J), no significant variations in stiffness were recorded, which is consistent with the absence of fiber breakage confirmed by CT imaging. These stiffness reductions are closely associated with the extent of the damage area, which increased proportionally with impact energy, as confirmed by ultrasonic testing. Furthermore, the depth of damage also increased with higher energy levels.

It was also noted that the maximum damage area determined by the ultrasonic method was consistently larger than the area measured via computed tomography (CT). This overestimation can be attributed to the inherent limitations of the ultrasonic technique, such as lower spatial resolution and sensitivity to delaminations, rather than to precise geometric quantification. The systematic overestimation observed in UT may be regarded as conservative from a safety standpoint, as it minimizes the risk of underestimating in-service damage. Nevertheless, such conservatism can be detrimental to maintenance optimization, potentially leading to unnecessary repairs or premature component replacement.

For the specimens used in this study, an impact energy of 15 J resulted in Barely Visible Impact Damage (BVID) in non-preloaded specimens. In tests involving combined loading—i.e., specimens preloaded statically and then dynamically—the extent of material damage depended significantly on the side from which the impact was applied. Specimens impacted on the compression side exhibited substantially more extensive damage than those struck from the tension side under the same conditions.

In practical applications, such as aircraft structural components (e.g., wing skins), where access is limited to one side and the exact impact point is unknown, identifying even structurally critical damage can be extremely challenging without specialized diagnostic tools. This underscores the need for advanced non-destructive testing (NDT) techniques and intelligent monitoring systems.

Additionally, it was observed that the contact time between the striker and the specimen varied depending on the static pre-stress configuration. Specifically, the contact time was shorter when the impact occurred on the tension side compared to the compression side. This reduction in contact duration under tensile preloading can be attributed to the decreased compliance of the specimen. Tensile pre-stress leads to partial straightening and stiffening of the fibers, which increases the effective structural rigidity and accelerates the rebound of the striker. In contrast, compressive preloading promotes local instability and microbuckling, thereby increasing the compliance of the structure and prolonging the impactor–specimen interaction. Analysis of the time–force curves obtained from the striker’s integrated sensor system enables both qualitative and partially quantitative assessment of the specimen’s structural condition.

Finally, the study confirmed the existence of threshold pre-stress values, beyond which the detrimental effects of impact loading markedly intensify. The most pronounced changes were observed for a pre-stress level of approximately 65% of the failure load, indicating that this threshold falls within the safety factors commonly adopted in commercial aircraft design (recommended value of 1.5 by the FAA). This finding suggests that the identified limit may serve as a useful reference for assessing safety margins in aerospace structures, supporting both the validation of current design practices and the development of more reliable inspection and maintenance strategies [43].

The results clearly indicate that even under relatively small bending loads, the fibers exhibit superior performance under tensile stresses, which translates into a greater resistance to compressive-type impacts. This asymmetry in the laminate response can be explained by the fundamentally different mechanisms governing damage initiation under tension and compression. Consequently, in the case of combined loading (preloading with a static component and subsequent impact), the material response depends strongly on whether the impacted component introduces additional tensile stresses or amplifies compressive stresses. When the impact load generates tensile stresses or reduces the magnitude of normal compressive stresses, the onset of damage is delayed, and a higher amount of energy is required to initiate failure. This situation was observed in variant I, where the dynamically loaded zone was pre-tensioned, and the subsequent impact reduced the effective tensile stress level. Conversely, in variant II, where the dynamically applied load acted directly on a pre-compressed zone, the impact caused an additional increase in compressive stresses, thereby intensifying the local compressive failure mechanisms. As a result, the experimental observations confirmed that specimens subjected to impact loading from the compressed side exhibited lower resistance to impact compared with those dynamically loaded on the tensile side. These findings are consistent with previous reports indicating that failure under compression is often dominated by fiber kinking, microbuckling, and matrix shear, whereas under tensile loading the primary mode of failure is fiber breakage, which typically requires higher energy for initiation. This behavior can be additionally explained by the so-called tensile straightening effect, whereby fibers subjected to tensile stresses tend to align and straighten, thus enhancing their load-carrying capacity and delaying the onset of damage [44].

The [0°]_25_ unidirectional layup employed in this study represents a highly anisotropic configuration and does not directly capture the response of aerospace-grade quasi-isotropic laminates. Its selection was motivated by the need to isolate the fundamental influence of pre-stress on the fiber-dominated response, enabling a clearer interpretation of preload–impact interactions. The intention was to establish a baseline case that can be extended to more complex stacking sequences. While it is recognized that multidirectional laminates exhibit distinct damage mechanisms and energy absorption characteristics, the overarching conclusion of this study—specifically, that pre-stress exerts a significant influence on impact-induced damage initiation and progression—is anticipated to remain valid for cross-ply and quasi-isotropic composites, although the specific damage morphologies and critical thresholds are expected to differ.

## 5. Conclusions

The results of this investigation have demonstrated that static preloading exerts a decisive influence on the impact response of unidirectional CFRP laminates subjected to low-energy impacts. The study provides both mechanistic insights into the initiation and propagation of damage and practical guidelines relevant to the design and certification of aerospace composite structures.

From a scientific perspective, the experiments revealed the strong dependence of damage initiation on impact energy. Barely Visible Impact Damage (BVID) was detected in non-preloaded laminates at an impact energy of 15 J. This threshold is particularly significant, as it was established for CFRP specimens with a thickness of 7.3 mm, which is greater than that of many laminates commonly employed in aerospace applications. This finding highlights that even low-energy impact events, such as accidental tool drops during maintenance operations, may result in structurally relevant damage and compromise the integrity of aircraft skin panels.

The presence of preloading was found to fundamentally alter the impact response, producing a highly asymmetric behavior, depending on whether the tensile or compressive side of the laminate was impacted. Under tensile preloading, shorter interaction times were observed, which can be attributed to reduced compliance and the partial straightening of fibers, leading to an increase in effective rigidity and accelerated rebound of the impactor. Conversely, compressive preloading promoted local instabilities and fiber microbuckling, thereby increasing compliance and prolonging the interaction. These observations not only provide mechanistic insight into the asymmetric impact behavior of CFRP laminates but also emphasize the need to incorporate time-dependent parameters into predictive models of impact response, particularly in the context of damage tolerance evaluation for preloaded aerospace structures.

The study further contributes to the theoretical understanding of critical pre-stress thresholds. At approximately 65% of the bending failure load, compressive-side preloading caused catastrophic damage even under relatively low impact energies. This observation indicates the existence of a critical limit that coincides with the safety margins conventionally employed in aerospace structural design, thereby strengthening the rationale for their continued adoption. These results enhance the broader understanding of how pre-stress asymmetry governs energy absorption and failure mechanisms in CFRP laminates.

From an engineering and application standpoint, the findings underline the necessity of explicitly considering both the direction and magnitude of pre-stress in the design and certification of composite structures. The heightened vulnerability of laminates subjected to compressive preloading underscores the importance of conservative safety factors and supports the need for the rigorous evaluation of structures exposed to combined loading scenarios during their operational lifecycle.

The comparative assessment of non-destructive testing (NDT) methods also yielded practically relevant conclusions. While computed tomography provided the most accurate characterization of internal damage, ultrasonic inspection consistently overestimated the damage extent. Nevertheless, due to its conservative character, lower cost, and ease of implementation, ultrasonic testing remains indispensable in aerospace maintenance, and its application should be complemented by advanced imaging methods in critical cases.

Taken collectively, the findings substantiate the asymmetric influence of tensile and compressive pre-stress on the impact response of unidirectional CFRP laminates and establish an experimental basis for the further development of predictive models describing damage initiation and progression. From the perspective of aerospace engineering, the results not only reinforce current safety standards but also indicate the potential for refining certification practices through explicit incorporation of preloading effects into structural assessment methodologies.

Future research should extend these investigations to multidirectional and quasi-isotropic laminates in order to achieve a more comprehensive representation of aerospace-grade composites under operational loading scenarios. Furthermore, the integration of experimental evidence with advanced computational modelling is expected to facilitate the development of robust predictive tools capable of bridging laboratory-scale investigations with full-scale structural performance, thereby enhancing the reliability of impact damage tolerance assessments in composite airframe structures.

## Figures and Tables

**Table 1:** Contact time between impactor and specimen depending on the static preload variant.

	Variant I	Variant II	Time Difference [%]
Average contact time [ms]	2.289 ± 0.075	3.348 ± 0.135	31.63 ± 5.52

## Data Availability

The original contributions presented in this study are included in the article. Further inquiries can be directed to the corresponding author.

## Abbreviations

The following abbreviations are used in this manuscript:

BVID	Barely Visible Impact Damage
CFRP	Carbon Fiber-Reinforced Polymer
CT	Computed Tomography
LVI	Low-Velocity Impact
NDT	Non-Destructive Testing
UT	Ultrasonic Testing
